# Circulating Tumour DNA in Muscle-Invasive Bladder Cancer

**DOI:** 10.3390/ijms19092568

**Published:** 2018-08-29

**Authors:** Melissa P. Tan, Gerhardt Attard, Robert A. Huddart

**Affiliations:** 1 Division of Radiotherapy & Imaging, Institute of Cancer Research, 15 Cotswold Road, Sutton, Surrey SM2 5NG, UK; melissa.tan@icr.ac.uk; 2 Academic Urology Unit, The Royal Marsden NHS Foundation Trust, Downs Road, Sutton, Surrey SM2 5PT, UK; 3 Research Department of Oncology, UCL Cancer Institute, University College London, 72 Huntley Street, London WC1E 6DD, UK; g.attard@ucl.ac.uk

**Keywords:** circulating tumour DNA (ctDNA), muscle-invasive bladder cancer (MIBC), biomarker

## Abstract

Circulating tumour DNA (ctDNA) is an attractive tool in cancer research, offering many advantages over tissue samples obtained using traditional biopsy methods. There has been increasing interest in its application to muscle-invasive bladder cancer (MIBC), which is recognised to be a heterogeneous disease with overall poor prognosis. Using a range of platforms, studies have shown that ctDNA is detectable in MIBC and may be a useful biomarker in monitoring disease status and guiding treatment decisions in MIBC patients. Currently, with no such predictive or prognostic biomarkers in clinical practice to guide treatment strategy, there is a real unmet need for a personalised medicine approach in MIBC, and ctDNA offers an exciting avenue through which to pursue this goal. In this article, we present an overview of work to date on ctDNA in MIBC, and discuss the inherent challenges present as well as the potential future clinical applications.

## 1. Introduction

In the drive towards personalised medicine, circulating tumour DNA (ctDNA) is an invaluable tool in cancer research, offering unique advantages over tissue samples collected using traditional biopsy methods. Its collection via a simple blood draw allows serial samples to be conveniently and safely taken over a course of treatment, thus facilitating the study of tumour dynamics, treatment resistance, and disease progression. Furthermore, it has been suggested that ctDNA samples are likely to provide a more representative snapshot of an individual’s cancer compared with biopsy samples as tumour clones from the primary, micro-, and macro-metastatic deposits are present in a single sample [1,2].

These advantages have been exploited in numerous studies across various cancers including colorectal, breast, prostate, and lung malignancies [3,4,5,6]. ctDNA levels have been shown to be associated with disease burden [7,8] and in an analysis of serial samples, increasing levels have been shown to pre-date radiological progression [4,9,10]. Analysis of sequential samples taken over a course of treatment have also demonstrated tumour evolution with the emergence of subclones documented at disease progression [11,12,13]. In addition to plasma, tumour DNA fragments have also been detected in other body fluids such as urine and cerebrospinal fluid [14].

There has been a surge of interest in recent years focusing on ctDNA in muscle-invasive bladder cancer (MIBC); a heterogeneous disease with an aggressive natural history and poor prognosis. With no predictive or prognostic biomarkers in current clinical practice to guide treatment strategy, there is a real need to develop a personalised medicine approach to optimise patient outcomes, and ctDNA offers an innovative approach to address this challenge. 

In this review article, we provide a brief background on ctDNA before summarising research to date on ctDNA in MIBC, discussing the challenges present and future clinical applications. 

## 2. Circulating Tumour DNA: Background

### 2.1. Biology of Circulating DNA

It has long been known that plasma contains nucleic acid fragments including those of DNA (genomic, mitochondrial, and viral), RNA, and micro-RNA; these have been collectively termed circulating nucleic acids. The mechanism by which nucleic acid fragments are released into the circulation remains under debate, but is thought to involve apoptosis, necrosis, and secretion [15]. Circulating DNA (cDNA) fragments are typically less than 200 bp in length. They are thought to undergo hepatic and renal excretion, and the reported half-life of cDNA fragments ranges between 16 min and 2.5 h [9,15,16]. While increased levels of cDNA are seen in malignancy and have been reported to be associated with tumour burden and prognosis in some cancer sites [17,18,19], raised levels are also seen in benign conditions such as pregnancy, trauma, or inflammation, meaning that cDNA levels alone are not necessarily a specific biomarker in the diagnosis or management of cancer [15]. 

In the majority of patients with malignancies, cDNA is mainly composed of wildtype, i.e., normal DNA, but may also contain fragments derived from the primary tumor, distant metastases, and micrometastases. The proportion of circulating tumour DNA (ctDNA) fragments (tumour fraction) has been shown to increase with disease burden [3,4,7], and also to vary between tumour types [7]. Although some patients with very advanced disease have demonstrated high tumour fractions above 50% [11], these are the minority and in numerous studies of metastatic disease, tumour fractions as low as 0.04% [4,20] have been reported. Indeed, a recent abstract reported an estimated median tumour fraction of 1.9% in metastatic urothelial cancer [21]. One of the challenges of working with ctDNA, therefore, is in detecting and quantifying the tumour fraction, particularly in the setting of early disease where levels may be in region of 0.01% [3].

### 2.2. Circulating DNA vs. Circulating Tumour DNA

In order to distinguish ctDNA from wildtype DNA, it is necessary to identify and detect somatic aberrations harboured by the tumour fragments. Quantification of fragments containing aberrations, which may include single nucleotide variants (SNVs), copy number alterations (CNAs), or structural variants, is used as a surrogate of tumour fraction. 

There are various approaches to achieve this. One strategy is to first identify aberrations present in a patient’s tumour tissue using either a broad de novo sequencing approach (e.g., whole exome or whole genome sequencing) or using a pre-determined set of assays or targeted sequencing panels encompassing known relevant aberrations in the cancer of interest. Aberrations identified in tumour tissue can then be detected in cDNA using polymerase chain reaction (PCR)-based specific assays or a focused next generation sequencing (NGS) approach. An alternative is to sequence the cDNA upfront using either of the approaches described above. However, in order to perform whole genome or whole exome sequencing on cDNA, a minimum tumour fraction is required, in the order of at least 10–20% [22,23], and so this approach is precluded in a significant proportion of cases where tumour fraction does not meet this threshold. 

While employing a broad sequencing approach allows an overview of aberrations present, allows assessment of copy number alterations, and supports the design of patient-specific plasma assays, cost may often be a prohibitive factor, particularly if high levels of coverage are sought to identify low frequency aberrations with confidence. 

A targeted panel or pre-determined set of assays is more cost-effective, but there is the risk that tumour fraction may be underestimated or ctDNA may not be detected if the individual’s relevant mutations are not included in the panel. Furthermore, using specific assays means that mutations arising over time or under selective pressure from treatment will not be identified unless included on the panel. There is thus a balance to be achieved in selecting an approach with sufficient breadth and depth that allows the question being asked to be answered. In the context of MIBC, we shall see that both approaches have been employed, and we discuss this further in the next section of this review. 

In trying to improve detection rates of ctDNA, it has been suggested that as tumour fragments are shorter than wildtype DNA fragments [24,25,26], fragment size selection will be enriched for ctDNA and thus allow very low frequency aberrations to be more readily detected [27]. This approach may be useful for increasing detection of ctDNA in patients with early disease or rare variants. 

Whichever approach is employed, ultra-sensitive techniques are required to detect the low levels of ctDNA present. Digital PCR techniques such as BEAMing or droplet digital PCR (ddPCR) have allowed research in this area to progress with sensitivity thresholds of 0.01% [3,28]. However, these approaches only allow a few aberrations to be interrogated at a time, and thus require a priori knowledge regarding aberrations to be detected.

## 3. Muscle-Invasive Bladder Cancer

### 3.1. Overview

Muscle-invasive bladder cancer is a heterogeneous disease with an overall poor prognosis [29]. Several molecular profiling studies have identified a number of molecular subtypes that are suggested to have different spectrums of mutations and clinical behaviour [30,31,32]. Sequencing studies have demonstrated that it has a high mutational burden, third only to melanoma and lung [30], but yet, in contrast to other tumour types, there are currently no approved biomarkers to guide its management. Radical treatment options in MIBC include neoadjuvant platinum-based combination chemotherapy followed by surgery, or in patients where surgery is deemed unsuitable or a bladder preservation strategy is being pursued, chemoradiation may be offered as part of a trimodality approach. In the palliative setting, platinum-based chemotherapy remains the mainstay of treatment, with the recently approved immune checkpoint inhibitors offering a further line of treatment. There is a real unmet clinical need for predictive and prognostic biomarkers in MIBC in order to develop a personalised approach if outcomes are to be improved. ctDNA in MIBC thus promises to be an exciting avenue to enable researchers to better understand the molecular biology, study treatment resistance and disease progression, and identify potential therapeutic targets.

### 3.2. Potential Clinical Applications of ctDNA in MIBC

ctDNA has the potential to be clinically useful at every step of the treatment pathway in MIBC, from early diagnosis, monitoring or predicting response to treatment in both the radical and palliative settings, assessing the need for adjuvant treatment, and in monitoring for recurrence or progression. Currently, one area of particular interest is in predicting and monitoring response to neoadjuvant chemotherapy. International guidelines [33,34] currently recommend all patients with localised disease are offered neoadjuvant cisplatin-based chemotherapy. However, up to 60% of patients do not respond [35,36] and these patients have thus not only been subjected to unnecessary toxicity, but have also experienced a delay to their definitive treatment, with a potential detrimental effect on outcome [37]. A minimally invasive biomarker to predict response or more sensitively monitor response would thus be of huge clinical benefit, and ctDNA offers the potential to achieve this.

## 4. CtDNA in MIBC

### 4.1. Overview

Some of the first work on ctDNA in MIBC dates back to 1991 when Sidransky et al. demonstrated the presence of *p53* mutations in the urinary sediment of three patients with MIBC [38]. Over a decade later, cDNA and ctDNA levels in plasma were shown to be higher in bladder cancer patients than in healthy controls [39,40]. Then, there followed a hiatus in publications on ctDNA in MIBC until Bettegowda et al.’s landmark paper [7], where next generation sequencing was performed on tumour tissue from three patients with metastatic MIBC as part of a broader pan-cancer cohort. A *p53* mutation was identified in each of the patients and was successfully detected in plasma in all three cases. In this small subset of patients, clinical outcomes were not reported, although the paper overall reported increased ctDNA levels with advanced disease. In the last two years, there has been a surge of publications looking at ctDNA in MIBC. Table 1 summarises representative publications including select poster abstracts as of June 2018. Some of the earlier studies have included superficial, i.e., <T2 disease; non-muscle-invasive bladder cancer (NMIBC), and MIBC in a cohort. However, more recently, MIBC has been considered separately and this is in keeping with fact that NMIBC and MIBC have been shown to have different molecular profiles [41]. 

### 4.2. ctDNA Is Detectable Using Commercially Available Panels 

In 2016, Sonpavde et al. [42] presented work showing that aberrations in cDNA could be detected in 25/29 (86.2%) patients with metastatic urothelial cancer using a commercially available panel composed of 68 cancer-related genes. Using the updated, now 73-gene panel (Guardant360), the group further went on to demonstrate aberrations in plasma from 265/294 (90%) patients with metastatic lower tract urothelial cancer [43]. *TP53* (48%), *ARID1A* (17%), and *PIK3CA* (14%) were the most commonly reported aberrations. They also compared these results from plasma with publically available data from previous NGS studies reporting aberrations in tumour tissue, and reported similar results in terms of the frequency of reported aberrations included on the panel. 

Using an alternative 62-gene panel (FoundationACT), McGregor et al. [21] found at least one aberration in plasma of 48/66 (73%) patients with metastatic urothelial cancer. A proportion of their cohort also had sequencing data on baseline tumour tissue available, and the authors reported an example where plasma taken at the time of cisplatin resistance showed persistence of *ERBB2* and *TP53* mutations identified in baseline tumour tissue alongside a new *NF1* aberration. This demonstrates a potential application of ctDNA in furthering our understanding of disease progression and treatment resistance in MIBC, with the potential ability to monitor patients during treatment for evidence of response or the emergence of new potential targets. 

Of note, both of these ctDNA panels that were used contained at most only 9 of the most frequent 23 gene mutations documented in the TCGA report [30], and omitted many of the chromatin-modifying gene alterations frequently seen in MIBC, for example, *KMT2D* and *KDM6A* (observed in 28% and 26% of TCGA MIBC cases, respectively). *ERCC2*, which has been put forward as a potential biomarker of sensitivity to cisplatin chemotherapy [44], is also absent. However, as the primary aim of these panels is to identify potential targeted therapy options in the clinical setting, it could be argued that these omissions do not have any clinical impact for MIBC patients, given that there are currently no associated therapies for these targets. However, in the research setting, the omission of these frequently mutated genes is a limitation of these panels in the exploration of potential targets and the study of disease biology. 

The high aberration detection rates of up to 90%, however, are not to be ignored as other groups using custom, albeit much smaller panels/assays have reported lower ctDNA detection rates. Of note, the above cohorts were composed exclusively of patients with advanced disease where ctDNA levels would be expected to be higher and thus more readily detectable. As yet in the literature, there are no reports of using such commercially available panels to profile patients with non-metastatic disease. 

### 4.3. Using Patient-Specific Assays to Detect ctDNA

#### 4.3.1. In NMIBC Cohorts

In one of the first papers to apply a personalised approach to bladder cancer, Birkenkamp-Demtröder et al. [45] used whole exome sequencing (WES), whole genome sequencing (WGS) and/or matepair sequencing to identify mutations in fresh frozen tumour tissue before designing personalised ddPCR assays for use on urine and plasma in a cohort comprising of 12 patients with NMIBC with either disease recurrence or progression to MIBC. ctDNA was detectable in 10/12 (83.3%) patients, including those with non-invasive disease only. In 4/6 (66.7%) patients progressing to muscle-invasive disease, detection of ctDNA pre-dated clinical diagnosis of MIBC by several months.

Christensen et al. [46] also detected ctDNA in patients with NMIBC and MIBC, but used a targeted approach with ddPCR assays to detect three hotspot mutations in *PIK3CA* (E545K) and *FGFR3* (S249C, Y373C), first in tumour tissue and then in plasma and urinary supernatant. In 201 urine samples from patients with NMIBC taken during their disease course, they reported overall higher urinary ctDNA levels in those later progressing to MIBC when compared with those with no progression. Kaplan-Meier progression free survival estimates for a subset of 25 showed that those with ctDNA urinary levels above the median at initial visit had increased progression rates to MIBC (7/13; 54%) compared with those with ctDNA levels below the median (1/12; 8%; *p* = 0.036).

However, within their cystectomy cohort of 468 patients, of whom at least 363 had MIBC, only 44/403 (11%; 65 excluded as insufficient material) had at least one mutation detected in tissue using the *PIK3CA* and *FGFR3* assays. Of those, 27 urine and 27 plasma samples were analysed. A third of patients (9/27) had detectable ctDNA in plasma. Increased ctDNA levels in plasma were associated with lower recurrence-free survival and overall survival. ctDNA levels were overall found to be higher in urine than in plasma [46].

Using the TCGA data portal [53], it can be shown that 62/412 (15%) MIBC patients possess at least one of the three hotspot mutations tested by Christensen et al. [46], which is slightly higher than the detection rate of 11% reported. The authors suggest that the procurement of tissue from a tissue microarray contributed to a low yield of DNA at the tumour tissue screening step, which may have resulted in missed cases. The subsequent low detection rate (33%) in plasma, despite the use of ddPCR, likely reflects the fact that as a cystectomy cohort, patients had localised disease with very low ctDNA fractions. This study highlights the importance of selecting aberrations to capture as many patients as possible, especially when utilising techniques where only a few aberrations can be interrogated at one time.

However, despite small numbers and heterogeneous cohorts consisting mainly of NMIBC, these studies demonstrate proof-of-concept in using both broad and targeted approaches in screening tumour tissue for aberrations to subsequently detect in plasma and urine in patients with bladder cancer. The results raise the possibility of using ctDNA to monitor patients with NMIBC for progression to MIBC. However much work is needed before this can be explored in the setting of a prospective clinical trial, and one of the key steps will be in determining the optimal aberration panel with which to identify and quantify ctDNA.

#### 4.3.2. In MIBC Cohorts

Building upon previous work, Birkenkamp-Demtröder et al. [49] used the same three ddPCR assays for *PIK3CA* and *FGFR3* hotspot mutations in combination with WES to screen diagnostic tumour tissue taken at transurethral resection (TUR) in 60 patients with MIBC, comprising of 50 commencing NAC and 10 commencing palliative chemotherapy. Using the three assays, at least one mutation was found in 19/60 patients (31.7%). WES was additionally performed on tissue from 24 patients and aberrations identified in 100%. The authors went on to design 84 personalised assays for 61 genes for a final cohort of 26 patients. Of note, only 2/26 (7.7%) had aberrations identified using only the *PIK3CA*/*FGFR3* assays. Plasma and urine samples were tested although longitudinal results were available only for plasma. Blood was taken at pre-defined time points during treatment and follow-up.

Of the 24 patients proceeding to radical cystectomy following chemotherapy, 12/24 (50%) relapsed at a median of 275 days. In 6/12 (50%) of relapsing patients, ctDNA was detectable at a median of 137 days resulting in a median positive lead time of 101 days. However, ctDNA was also detected at some time point in 50% of patients who remained disease-free post-surgery, so the presence of ctDNA post-cystectomy is not specific for relapse [49]. However, the authors noted a significant association between high plasma ctDNA levels in samples taken at one week to four months post-cystectomy, and disease relapse, thus suggesting that ctDNA may allow more sensitive detection of disease recurrence post-surgery, and may be useful in the selection of patients for further treatment. Samples taken before, during, and after treatment for disease relapse were also analysed with an overall decrease in levels after 2–5 cycles of treatment correlating with radiological response, and subsequent increase in levels correlating with progression. 

While this paper omits some technical details from its methodology, it sets the scene for the potential use of ctDNA in the post-operative setting to assess risk of recurrence and perhaps guide decisions on adjuvant treatment. Once again, the importance of selecting an appropriate panel of aberrations to target is highlighted, given that in 92.3%, personalised assay design was dependent upon data from whole exome sequencing.

The potential for ctDNA to detect recurrence before clinical or radiological confirmation was also demonstrated by Patel et al. [48]. In a cohort of 17 MIBC patients embarking on neoadjuvant platinum-based chemotherapy, the authors performed tagged amplicon sequencing (TAm-Seq) using a bladder cancer-specific panel of eight genes to detect mutant DNA in TUR tumour tissue, plasma, urinary cell pellet (UCP), and urinary supernatant (USN). The eight genes were *BRAF*, *CTNNB1*, *FGFR3*, *HRAS*, *KRAS*, *NFE2L2*, *PIK3CA*, and *TP53*, and were anticipated to encompass 72% of patients based upon TCGA data. A *TERT* promoter assay did not perform well and so was excluded. They also performed shallow whole genome sequencing in order to assess copy number alterations (CNAs). Samples were collected over a median period of 83 days from commencing NAC, with a median of 15 samples per patient. Patients were followed up for a median of 742 days from commencing NAC, and 588 days after completing definitive therapy.

On sequencing the available tumour tissue from 16 patients, single nucleotide variations (SNVs) were detected in 12/16 (75%) patients. The most frequent SNVs were in *TP53*, *KRAS*, and *PIK3CA*. Copy number alterations were identified in all 16 TUR samples, with the most frequent being *CDKN2A* loss, *E2F3/SOX4* gain, and *PPARG* gain. 

Subsequent testing in plasma, UCP, and USN in the 12 patients with tumour tissue SNVs showed detection of mutant DNA in 4/12 (33%), 5/12 (42%), and 5/12 (42%), respectively. CNAs were detected in 4/16 (33%), 8/15 (53%), and 8/16 (50%) of plasma, UCP, and USN samples, respectively. Of note, shallow WGS on serial samples from five patients showed evidence of tumour evolution under the selective pressure of NAC. Overall, aberrations were detected in 10/17 (59%) patients in plasma and urine samples taken prior to commencing NAC. Detection of aberrations at this point did not predict the response to treatment. However, upon analysing samples taken prior to cycle two NAC, the authors found that mutant DNA was present in 5/6 (83%) patients that relapsed, but was not detected in relapse-free patients (specificity 100%, sensitivity 83%). The median lead time over radiological diagnosis of progression was 243 days. Of note, of the five patients with mutant DNA present, only one patient had aberrations detected in plasma. Overall, higher levels of detection were noted in urine compared with plasma, although no single sample type captured all the aberrations present [48].

Although this was a relatively small cohort, the comprehensive assessment of plasma and urine shows great promise for ctDNA as a potential biomarker of response to treatment in the radical setting, and suggests that assessment of both plasma and urine is warranted at least in the neoadjuvant setting. In both these studies, it is again demonstrated that the optimal panel of genes and platform to interrogate MIBC remains unclear with *PIK3CA* and *FGFR3* assays allowing ctDNA analysis in only 7.7%, and a combination of eight TAm-Seq assays and shallow WGS detecting ctDNA in 59%. However, with sequencing costs continuing to fall, it may be that broad approaches such as WES, which identified aberrations in 100% of tumour tissue samples, become more accessible. This strategy, however, would depend upon the availability of contemporary tissue samples and would thus likely necessitate repeat biopsies, particularly in those previously treated or with relapsed disease, which may be neither achievable on a practical level nor acceptable to patients.

Another question to consider is whether the identification of aberrations in tumour tissue first is necessary or indeed useful given potential intra-tumour heterogeneity and tumour evolution over time. Cheng et al. [52] used the 341–468 gene MSK IMPACT panel to profile plasma samples from 26 patients with metastatic urothelial cancer. At least one mutation was detected in 18/26 (69%). For 15 patients, archived tumour tissue was also sequenced using the same panel. The interval between tissue and plasma sampling ranged from 35 days to >4 years, and 11/15 patients had received treatment during this period. They reported that the tissue and plasma profiles were identical in only 3/15 (20%) of patients where the interval between samples ranged from 35 days to <1.5 years. Six out of fifteen (40%) had mutations identified in plasma, but not in tissue, and vice versa in 11/15 (73%). They concluded that the differences may reflect tumour evolution or intratumour heterogeneity. It may then be that sequencing archived tissue to detect aberrations of interest may not always identify the most appropriate targets in ctDNA, and may not be the ideal strategy particularly in patients who have received treatment or demonstrated a change in disease status in the intervening period. In these situations, upfront analysis of ctDNA is an attractive option, particularly when repeat up to date tissue biopsies are not possible.

### 4.4. Using a MIBC-Specific Panel to Sequence Plasma Upfront

Vandekerkhove et al. [47] designed a 50-gene bladder cancer-specific panel based upon published data on recurrent mutations and copy number changes in bladder cancer, including the TCGA report. In designing the panel, the authors noted that 98% of patients from the TCGA MIBC dataset (consisting primarily of subjects with non-metastatic disease) had a non-synonymous mutation in at least one of the 50 genes included on their panel. With target depth of 500–1000×, aberrations would be detected in those with tumour fraction of 5% or more.

Fifty-one patients with MIBC were recruited, including 37 with nodal or distant metastases. Plasma from all those with nodal or distant metastases, and seven with organ-confined disease were sequenced using the targeted panel. Overall, 25/44 (56.8%) patients demonstrated a ctDNA fraction above the 2% detection threshold set. The tumour fraction ranged from 3.9 to 72.6% with all samples demonstrating greater than 30% tumour fraction originating from patients with distant metastatic disease. In those with tumour fractions between 3.9–30%, 52% had distant metastatic disease. Only one of the seven patients with localised disease had detectable ctDNA [47]. This association of higher tumour content with higher disease burden is in keeping with previous work in other tumour types [7]. In three patients with metastatic disease where ctDNA was detected at more than one time-point over the course of chemotherapy, aberrations identified were consistent [47]. Frequently mutated genes included *TP53*, *PIK3CA*, and *ARID1A*; over 50% of patients had chromatin-modifying gene aberrations and this work has demonstrated that like the commercially available panels, upfront NGS analysis of plasma cDNA can identify potentially actionable aberrations. Of note, the only recurrent mutations seen were known hotspot regions in *ERBB2*, *PIK3CA*, and the *TERT* promoter. All other mutations were unique to individual patients and this highlights the incredible (and challenging) heterogeneity seen in MIBC.

Whole exome sequencing was also performed on 11 samples with tumour fraction over 25% from eight patients, with the primary aim of comparing mutation rates derived from the targeted sequencing data with that from WES data. The WES results correlated with targeted sequencing data, although the difference in sequencing depth meant that mutations seen on targeted panel were not always called on exome data [47]. Mutation rates derived from whole exome data and targeted sequencing data were also correlated, which is of interest given that mutational burden has been put forward as a predictor of response to immune checkpoint agents. The potential to assess mutational burden on a plasma sample rather than tissue biopsy is a potential advantage in the clinical trial setting, where patients may not otherwise be able or willing to undergo an invasive procedure as part of trial entry.

## 5. Conclusions

In the last two years, significant progress has been made in the field of ctDNA in MIBC and the knowledge base has grown rapidly. We have seen that ctDNA can be detected, with varying degrees of sensitivity, in the plasma and urine of patients with localised and metastatic MIBC. The levels detected have been demonstrated to be associated with tumour burden and while samples taken at one time-point are able to allow the identification of potentially actionable aberrations, the real value of ctDNA lies in the ease of obtaining sequential samples. By analysing samples taken over a course of treatment or during follow-up, early results in small trials suggest that the presence of ctDNA may indicate minimal residual disease following surgery, or predict for future disease recurrence with greater sensitivity than that offered by current standard radiological assessment. This is hugely exciting and the implications are potentially practice changing, but there is much work to be done before ctDNA can be applied in the clinical setting.

A key challenge is in refining the detection of ctDNA. MIBC is somewhat unique from other cancer types where ctDNA research is perhaps more established. Whereas a select few assays, for example, *APC* or *KRAS* mutations in colorectal cancer, *BRAF* in melanoma encompass a significant proportion of patients, and are thus reliable aberrations to use a surrogate for tumour fraction, the equivalent targets have yet to be demonstrated in MIBC. While this is in part because of the heterogeneity of the disease, it is also likely attributable to the fact that the molecular landscape of MIBC was only more recently explored when compared with other cancer subtypes. It has since been put forward that 90% MIBC patients have at least one mutation in hotspot regions of *PIK3CA*, *TP53*, or the *TERT* promoter, and so a panel encompassing these should be of relevance to the vast majority [23]. While these genes were included in the 50-gene bladder cancer specific panel by Vandekerkhove et al. [47], the detection threshold set of 2% for tumour fraction likely accounts for their plasma ctDNA detection rate of 56.8% falling short of the theoretical 90% described. It will be of great interest to see whether more sensitive methods, for example, ddPCR assays for this three-gene panel, will indeed encompass the majority of MIBC patients, including those with localised disease where low tumour fractions make detection more technically challenging. Furthermore, the use of so-called molecular barcodes, as recently explored in MIBC samples, offer another method to improve detection thresholds [54].

A unique feature of bladder cancer is the availability of ctDNA in urine. We have seen that ctDNA was more readily detectable in urine in a cohort of patients undergoing neoadjuvant treatment [48], and it seems reasonable to suggest that this is by virtue of the close proximity of the primary tumour to urine, i.e., the shedding of tumour DNA fragments directly into urine. It may well be that urinary ctDNA is most relevant in patients with localised disease, while plasma ctDNA reflects the systemic burden of disease.

While great promise is shown in the use of ctDNA to detect recurrence earlier than current standard approaches, an important consideration is also whether or not detecting recurrence earlier has any impact on clinical outcomes, and this can only be determined through prospective clinical trials.

MIBC is rapidly catching up with other more established tumour sites in the field of ctDNA research, and the clinical implications are huge in this poor prognosis disease where there are currently no biomarkers in everyday clinical use. By fully harnessing the potential of ctDNA, a truly personalised approach bypassing spatial and temporal barriers in cancer research appears possible and is key in furthering our understanding of MIBC and ultimately improving clinical outcomes.

## Figures and Tables

**Table 1:** Representative publications including select poster abstracts as of June 2018.

Reference	Year	*n*	Cohort	Method	Key Findings
Bettegowda et al. [7]	2014	3	Metastatic MIBC	One-hundred-gene panel on tumour tissue; SafeSeq on plasma for patient-specific aberrations	*TP53* mutations detected in tumour tissue of 3/3 patients, and detectable in plasma of all three patients
Sonpavde et al. ^$^ [42]	2016	29	Advanced urothelial cancer (MIBC = 27/29)	Sixty-eight-gene commercially available panel to sequence a single plasma sample from each patient (Guardant360)	Aberrations detected in 86.2% patients
Birkenkamp-Demtröder et al. [45]	2016	12	NMIBC: six with recurrence and six with progression to MIBC	WES/WGS/mate-pair sequencing on tumour tissue; personalised ddPCR on sequential plasma samples	ctDNA detectable in 10/12; ctDNA detected several months before clinical diagnosis of progression to MIBC in 4/6 patients
Christensen et al. [46]	2017	1: 363; 2: 468	1: NMIBC 2: Cx (MIBC ≥ 363/468)	ddPCR assays to screen for *PIK3CA* and *FGFR3* hotspots in tissue, urinary supernatant, and plasma	Eleven percent of Cx cohort had ≥1 mutation detected in tumour tissue. Analysis of 23 paired urine and plasma showed higher levels of ctDNA in urine. In 27 Cx plasma samples analysed, high levels of ctDNA in plasma associated with disease recurrence
Vandekerkhove et al. [47]	2017	51	MIBC: 14/51 N0M0 disease; 27/51 N+ve/M1 disease	Bladder cancer-specific targeted panel (50 genes) on plasma from 44 patients including sequential samples; WES on plasma from eight patients to assess mutational burden	ctDNA detected in 25/44 (56.8%) patients with tumour fractions ranging from 3.9–72.6%; All with tumour fraction >30% had distant metastatic disease. Mutational burden derived from targeted sequencing panel consistent with that from WES
Patel et al. [48]	2017	17	MIBC (starting NAC)	Eight-gene TAm-Seq panel (for SNVs) and shallow WGS for copy number assessment on tumour tissue, plasma, urinary cell pellet, and urinary supernatant	Aberration detected in plasma or urine of 10/17 patients pre-NAC. Greater levels of ctDNA detection in urine. Detection of plasma or urine ctDNA pre-cycle two NAC associated with disease recurrence
Birkenkamp-Demtröder et al. [49]	2017	60	MIBC: 50 NAC; 10 palliative chemotherapy	Three ddPCR assays to screen for *PIK3CA* and *FGFR3* mutation in tumour tissue; WES on tumour tissue and germline in 24. Personalised ddPCR assays in plasma for 26 patients	*PIK3CA/FGFR3* assays positive in 19/60; ctDNA detectable in patients prior to clinically detected recurrence with median lead time 101 days
McGregor et al. ^$^ [21]	2018	66	Metastatic urothelial cancer	Commercially available 62-gene panel to sequence plasma (FoundationACT)	ctDNA aberrations detected in 48/66 (73%); Estimated median tumour fraction 1.9%
Barata et al. [50]	2017	22	Metastatic urothelial cancer	Compared sequencing results from tumour tissue and plasma sequenced using two different commercially available panels	Concordance between the two tests was 16.4%
Soave et al. [51]	2017	72	Radical Cx (>46/72 MIBC)	Tested 43 regions covering 37 genes for copy number variations (multiplex ligation dependent probe amplification)	cDNA had CNV in 48.6% samples; Overall CNV status not associated with clinical outcome; gain in *KLF5*, *ZFHX3*, and *CDH1* associated with reduced cancer-specific survival
Agarwal et al. [43]	2018	369	Metastatic urinary tract cancer (294/369—lower urinary tract cancer)	Commercially available 73-gene panel (Guardant360) used to sequence plasma	Similar aberrations seen when compared with publically available NGS data on tumour tissue
Cheng et al. ^$^ [52]	2017	26	Metastatic urothelial cancer	Used a 341–468-gene NGS assay (MSK-IMPACT) to sequence plasma (*n* = 26) and archival tumour tissue (*n* = 15)	ctDNA detected in 69% patients. Interval between plasma sampling and tissue collection was 35 days to >4 years; Identical tissue and plasma profiles in 20% (3/15)

Abbreviations: MIBC: muscle-invasive bladder cancer; NMIBC: non-MIBC; ctDNA: circulating tumour DNA; NAC: neoadjuvant chemotherapy; WES: whole exome sequencing; WGS: whole genome sequencing; Cx: cystectomy; NGS: next generation sequencing; CNV: copy number variation; SNV: single nucleotide variation; ddPCR: droplet digital polymerase chain reaction; N+ve: node positive; M1: distant metastases; TAm-Seq: tagged amplicon sequencing. ^$^: poster abstract.

## Abbreviations

bp	Base pairs
cDNA	Circulating DNA
ctDNA	Circulating tumour DNA
CNA	Copy number alteration
MIBC	Muscle-invasive bladder cancer
NAC	Neoadjuvant chemotherapy
NGS	Next generation sequencing
PCR	Polymerase chain reaction
TUR	Transurethral resection
SNV	Single nucleotide variant
WES	Whole exome sequencing
WGS	Whole genome sequencing
